# 椎旁阻滞对肺癌根治术患者术后镇痛效果及外周血肿瘤标志物水平的影响

**DOI:** 10.3779/j.issn.1009-3419.2015.02.10

**Published:** 2015-02-20

**Authors:** 冀衡 陈, 云霄 张, 川 黄, 克能 陈, 梦颖 范, 志毅 范

**Affiliations:** 1 100142 北京，北京大学肿瘤医院暨北京市肿瘤防治研究所麻醉科 Key Laboratory of Carcinogenesis and Translational Research (Ministry of Education), Department of Anesthesiology, Peking University Cancer Hospital and Institute, Beijing 100142, China; 2 100142 北京，北京大学肿瘤医院暨北京市肿瘤防治研究所胸外一科 Key Laboratory of Carcinogenesis and Translational Research (Ministry of Education), Department of Thoracic Surgery, Peking University Cancer Hospital and Institute, Beijing 100142, China

**Keywords:** 椎旁神经阻滞, 肺肿瘤, 肺叶切除术, 肿瘤标志物, Thoracic paravertebral block, Lung neoplasms, Lobectomy, Tumor marker

## Abstract

**背景与目的:**

围术期疼痛质量管理与肿瘤患者的预后相关，优化围术期镇痛方法，进而减轻围术期应激反应，减少阿片类药物用量从而减轻甚至避免由此引起的全身性不良反应和肿瘤标志物水平升高。肺癌患者血清中肿瘤标志物水平与肿瘤发展密切相关，国内外文献未见报道区域阻滞对肺癌肿瘤标志物的影响。本研究评价胸椎旁神经阻滞对胸腔镜肺癌根治术患者术后镇痛效果及外周血肿瘤标志物水平的影响。

**方法:**

采用随机数字表法将患者分为两组（各20例）：单纯全麻组（G组）和胸椎旁神经阻滞（paravertebral block, PVB）复合全麻组（GP）组。G组采用静脉诱导，静吸复合麻醉维持; GP组诱导前患者清醒时行PVB，PVB效果通过测定阻滞范围来判断，PVB起效后静脉诱导，静吸复合麻醉维持。两组均于术毕缝皮时启动静脉镇痛泵行患者自控静脉镇痛（patient controlled intravenous analgesia, PCIA）。分别于术后2 h、24 h和48 h行视觉模拟评分（visual analogue scale, VAS），记录按压次数以及镇痛药用量。分别于术前及术后24 h取两组患者的静脉血样本，检测肿瘤标志物癌胚抗原、糖链抗原199、糖链抗原125、神经元特异性烯醇化酶、细胞角质蛋白19片段的浓度。

**结果:**

择期行胸腔镜肺癌根治术患者40例，性别不限，年龄20岁-70岁，体重指数（body mass index, BMI）18 kg/m^2^-25 kg/m^2^，美国麻醉医师协会（American Society of Anesthesiologists, ASA）分级Ⅰ级或Ⅱ级。术后2 h，GP组患者的VAS评分明显低于G组（*P*=0.013）; 术后24 h，GP组患者的VAS评分也明显低于G组（*P*=0.025）; GP组术后24 h、48 h启动自控按钮次数明显少于G组（*P*值分别为0.021、0.026）; GP组24 h、48 h PCIA泵的输注总量明显低于G组（*P*值分别为0.006、0.011）。术后24 h，两组患者肿瘤标志物水平与术前比较变化不大（*P* > 0.05）; 比较两组患者肿瘤标志物水平，手术前后的差值差异无统计学意义（*P* > 0.05）。

**结论:**

胸椎旁神经阻滞可明显减轻胸腔镜肺癌根治术患者术后疼痛并减少静脉镇痛泵内阿片类药物用量; 胸椎旁神经阻滞对胸腔镜肺癌根治术患者肿瘤标志物水平无明显影响。

## Abstract (en)

**Background and objective:**

Perioperative management of pain associated with the prognosis of cancer patients. Optimization of perio-perative analgesia method, then reduce perioperative stress response, reduce opioiddosage, to reduce or even avoid systemic adverse reactions and elevated levels of tumor markers. Serum levels of tumor markers in patients with lung cancer are closely related to tumor growth. Clinical research reports on regional anesthesia effect on tumor markers for lung cancer are still very little in domesticliterature. The aim of this study is to evaluate the effects of thoracic paraverte-bral block on postoperative analgesia and serum level of tumor marker in lung cancer patients undergoing video-assisted thoracoscopic surgery.

**Methods:**

Lung cancer patients undergoing video-assisted thoracoscopic surgery were randomly divided into 2 groups (*n*=20 in each group). The patients in group G were given only general anesthesia. The thoracic paravertebral blockade (PVB) was performed before general anesthesia in patients of group GP. The effect of PVB was judged by testing area of block. Patient controlled intravenous analgesia (PCIA) pump started before the end of surgery in 2 groups. Visual analogue scale (VAS) score was recorded after extubation 2 h (T1), 24 h (T2) and 48 h (T3) after surgery and the times of PCIA and the volume of analgesic drugs used were recorded during 48 h after surgery. The serum levels of carcino-embryonic antigen (CEA), carbohydrate antigen 199 (CA199), carbohydrate antigen 125 (CA125), neuron-specific enolase (NSE), cytokeratin 19 fragment (CYFRA21-1) and squamous cell carcinoma (SCC) in 40 lung cancer cases undergoing video-assisted thoracoscopic lobectomy were measured before operation and 24 h after operation.

**Results:**

Forty American Society of Anesthesiologists (ASA) physical status Ⅰ or Ⅱ patients, aged 20 yr-70 yr, body mass index (BMI) 18 kg/m^2^-25 kg/m^2^, scheduled for elective video-assisted thoraeoscopic lobectomy, VAS scores at T1 and T2 were lower in group GP than those in group G (*P*=0.013, *P*=0.025, respectively), PCIA times during postoperative analgesia 24 h and 48 h were lower in group GP than those in group G (*P*=0.021, *P*=0.026, respectively), analgesic volume used during postoperative analgesia 24 h and 48 h were lower in group GP than those in group G (*P*=0.006, *P*=0.011, respectively). The level of tumor marker at post-operative were not significantly decreased than preoperative in both groups (*P* > 0.05).

**Conclusion:**

Patients in group G feel more painful and a higher dosage of dezocine is required to relieve the pain than group GP. Thoracic paravertebralblock has no influence on serum level of tumor marker in lung cancer patients undergoing video-assisted thoraeoscopic lobectomy.

目前肺癌是我国发病率与死亡率居首位的恶性肿瘤。手术仍是有可能治愈肺癌的主要手段。围术期镇痛和麻醉质量管理与术后恢复密切相关，甚至有研究认为其与肿瘤的长期预后相关^[1]^。目前，静吸复合全麻是肺癌手术的主要麻醉方法，如何优化麻醉镇痛方法，减轻围术期应激反应，减少阿片类药物用量从而减轻甚至避免由此引起的全身性不良反应是麻醉和外科关注的重要问题。恶性肿瘤的发生、发展与机体的免疫功能，尤其是与细胞的免疫功能减退有关。区域麻醉能够降低患者手术应激反应^[2]^，从而减轻由于应激反应所致的免疫抑制。肺癌血清中肿瘤标志物水平与肿瘤密切相关^[3]^，国内外文献未见报道区域阻滞对肺癌肿瘤标志物的影响。本研究旨在评价胸椎旁神经阻滞对胸腔镜肺癌根治术患者术后镇痛效果及外周血肿瘤标志物水平的影响，从而为肺癌手术患者临床麻醉方法的选择提供参考。

## 1 资料与方法

### 1.1 一般资料

本研究经北京大学肿瘤医院医学伦理委员会审查批准（审批号：2013121005），所有参加研究的患者均书面知情同意。择期全麻下行胸腔镜肺癌根治术患者，均经组织学或细胞学确诊为肺癌。术前排除伴有影响血中肿瘤标志物水平的情况（如炎症性疾病、肝肾疾病、妊娠及1个月内有手术史或创伤史）的患者，术前未做放化疗，无滥用酒精、药物、麻醉药物。共40例符合入选标准，其中男性23例，女性17例; 年龄20岁-70岁，体重指数（body mass index, BMI）18 kg/m^2^-25 kg/m^2^，美国麻醉医师协会（American Society of Anesthesiologists, ASA）Ⅰ级或Ⅱ级。腺癌28例，鳞癌12例。Ⅰa期8例，Ⅰb期13例，Ⅱa期15例，Ⅱb期4例。采用随机数字表法，将患者分为两组（各20例）：单纯全麻组（G组）和胸椎旁神经阻滞（paravertebral block, PVB）复合全麻组（GP）组。

### 1.2 方法

两组患者均接受静-吸复合全醉。麻醉诱导：静注舒芬太尼0.4 μg/kg、丙泊酚1 mg/kg-2 mg/kg、罗库溴铵1 mg/kg。术中吸入0.8-1 MAC七氟醚维持麻醉，氧流量1 L/min，间断静脉推注阿曲库铵25 mg，瑞芬太尼术中镇痛，术中根据患者血流动力学变化调整瑞芬太尼血浆靶浓度。术毕缝皮时开启患者自控静脉镇痛（patient controlled intravenous analgesia, PCIA）（AutoMed 3300，韩国），配方为：地佐辛0.8 mg/kg+盐酸托烷司琼0.2 mg/kg+生理盐水稀释至100 mL。设置负荷剂量2 mL，单次自控剂量2 mL，背景输注1 mL/h，锁定时间15 min。术后患者根据视觉模拟评分（visual analogue scale, VAS）自行调控PCIA泵：GP组患者在全麻诱导前侧卧位，手术侧在上，给予术侧PVB，选择T_4_-T_7_棘突向术侧旁开2.5 cm为穿刺点，用长10 cm的22号硬膜外穿刺针，在矢状面上稍向头侧方向刺入皮肤，当穿刺针触及横突时，将针退至皮肤水平，并向上调整穿刺进针方向10°-15°，将穿刺针滑过胸椎横突后，当穿刺至椎体横突下缘的肋横突韧带时，针尾连接无阻力的注射器，并试推有阻力感，穿刺针进入肋横突韧带后阻力增加，继续进针阻力消失时表明椎旁间隙穿刺成功。回吸无血、脑脊液或气即可注射局麻药液^[4]^，单次注入0.375%盐酸罗哌卡因每点5 mL。20 min后检测阻滞平面，确认阻滞成功。术前指导患者使用PCIA泵及VAS评分。

### 1.3 观测指标

记录术中瑞芬太尼用量; 术后2 h、24 h、48 h随访并记录VAS评分; 记录术后24 h和48 h启动自控按钮次数及输注总量; 术前及术后24 h取两组患者的静脉血样本，送北京大学肿瘤医院检验科检测肿瘤标志物癌胚抗原（carcino-embryonic antigen, CEA）、糖链抗原199（carbohydrate antigen 199, CA199）、糖链抗原125（carbohydrate antigen 125, CA125）、神经元特异性烯醇化酶（neuron-specific enolase, NSE）、细胞角质蛋白19片段（cytokeratin 19 fragment, CYFRA21-1）和鳞状细胞癌抗原（squamous cell carcinoma, SCC）的浓度。

### 1.4 统计学方法

采用SPSS 17.0统计学软件进行分析。计量资料以均数±标准差（Mean±SD）表示，组间比较采用单因素方差分析，组内比较采用重复测量设计的方差分析，计数资料比较采用χ^2^检验，*P* < 0.05为差异有统计学意义。

## 2 结果

### 2.1 两组患者一般资料和手术时间的比较

两组患者一般情况和手术时间的差异无统计学意义（*P* > 0.05），见表 1。

**1:** 两组患者一般资料和手术时间的比较 Clinical data and length of procedure of two groups

Group	*n*	Male/Female	Age	BMI (kg/m^2^)	ASA (Ⅰ/Ⅱ)	Length of procedure (min)
G	20	12/8	55±11	23±5	6/14	159±22
GP	20	11/9	53±12	22±4	7/13	162±21
BMI: body mass index; ASA: American Society of anesthesiologists; The patients in group G were given only general anesthesia. The thoracic paravertebral blockade (PVB) was performed beforegeneral anesthesia in patients of group GP.						

### 2.2 两组患者术中瑞芬太尼用量比较

两组患者术中瑞芬太尼用量差异（G组：1.23±0.56 *vs* GP组：1.21±0.62）无统计学意义（*P*=0.095）。术后第2 h，GP组患者的VAS评分明显低于G组（*P*=0.013）; 术后第24 h，GP组患者的VAS评分也明显低于G组（*P*=0.025）; 而术后第48 h，GP组患者的VAS评分与G组相比，差异无统计学意义（*P* > 0.05）。GP组术后24 h、48 h启动自控按钮次数明显少于G组（*P*值分别为0.021、0.026）; GP组24 h、48 h PCIA泵的输注总量低于G组（*P*值分别为0.006、0.011），见表 2-表 3。术后24 h，两组患者肿瘤标志物水平与术前比较变化不大，比较两组手术前后的差值差异无统计学意义（*P* > 0.05），见表 4。

**2:** 两组患者术后不同时点VAS评分比较 The VAS score of two groups postoperatively

Group	*n*	Postoperative 2 h	Postoperative 24 h	Postoperative 48 h
G	20	4.0±1.7	2.1±0.5	1.0±0.6
GP	20	0.8±0.7^a^	0.6±0.4^b^	0.7±0.4
VAS: visual analogue scale. Compared with group G, ^a^*P*=0.013, ^b^*P*=0.025.				

**3:** 两组患者术后启动自控按钮次数和输注总量比较 The total number of attempts and analgesic dosage on PCIA pump of two groups postoperatively

Group	*n*	Number of attempts			Analgesic dosage	
		24 h	48 h		24 h	48 h
G	20	12.0±5.5	16.7±6.2		50.2±6.7	81.5±7.5
GP	20	3.1±1.6^a^	5.2±2.7^b^		29.5±6.5^c^	60.3±6.7^d^
Compared with group G, ^a^*P*=0.021, ^b^*P*=0.026, ^c^*P*=0.006, ^d^*P*=0.011. PCIA: patient controlled intravenous analgesia.						

**4:** 两组患者手术前后血清肿瘤标志物水平比较 Compared with two groups serum level of tumor marker in preoperative and postoperative

Tumor marker	Preoperative			Postoperative 24 h	
	G	GP		G	GP
CEA (ng/mL)	3.15±1.68	2.93±1.01		3.19±1.35	2.88±0.51
CA199 (U/mL)	14.08±10.41	13.92±9.58		15.63±11.62	13.69±8.25
CA125 (U/mL)	13.61±10.87	13.29±9.17		12.75±8.33	13.35±7.88
NSE (ng/mL)	17.21±13.69	16.56±10.83		18.68±11.33	15.89±7.88
CYFRA21-1 (ng/mL)	2.73±1.29	2.71±1.06		2.84±1.19	2.63±1.01
SCC (ng/mL)	1.91±1.84	2.06±1.65		1.88±1.19	1.96±1.36
CEA: carcino-embryonic antigen; CA199: carbohydrate antigen 199; CA125: carbohydrate antigen 125; NSE: neuron-specific enolase; CYFRA21-1: cytokeratin 19 fragment; SCC: squamous cell carcinoma.					

### 2.3 两组术后不良反应比较

G组术后嗜睡发生2例（10%），GP组术后嗜睡发生1例（5%）; G组术后恶心、呕吐发生1例（5%），GP组术后恶心、呕吐发生1例（5%）; 两组无一例患者出现呼吸抑制，差异均无统计学意义（*P* > 0.05）。

## 3 讨论

目前肺癌为多发疾病，且肺癌患者易发生转移，如脑转移^[5]^等。癌症患者普遍存在免疫功能受损，围术期疼痛和大量阿片类药物应用等因素可加重患者的免疫功能损害，对恶性肿瘤的复发和转移造成不利影响。如果将区域麻醉与全身麻醉联合应用，可以大大减少阿片类药物和吸入麻醉药的用量，从而保护肿瘤患者免疫功能，有利于术后恢复，减少术后复发^[1]^。

研究^[6]^发现肿瘤标志物对于肺癌的诊断、手术疗效、预后评估、病情监测等有一定临床价值。NSE、CYFRA21-1、CA199、CA125、CEA是临床上应用较多的肺癌肿瘤标志物。临床采用电化学发光法检测肺癌患者血中CEA、CA199、CA125、NSE、CYFRA21-1的含量。结果肺癌患者血清CEA、CA125、CA199、NSE、CYFRA21-1的含量均明显高于正常人和良性肺疾病患者; 在多种标志物联合检测中，这5种标志物联合检测CEA+CA125+CYFRA21-1+CA199+NSE的敏感性最高^[7]^。研究^[8]^发现，肺癌细胞能直接产生CEA，其作为肺癌标志物的灵敏度为35%-77%。Niklinski等^[9]^研究显示CA125是肺腺癌的标志物。测定肺癌患者CA125水平可为临床治疗提供重要依据，有利于对肺腺癌的诊断。NSE是一类糖酵解酶，主要存在于神经元及外周神经内分泌组织中，小细胞肺癌是具有神经分泌特性的肿瘤，因此，NSE有助于小细胞肺癌的诊断及其与非小细胞肺癌的鉴别诊断，目前已公认为NSE可作为小细胞肺癌高特异性、高灵敏性的肿瘤标志物^[10]^。CYPRA21-1是细胞结构蛋白，一般位于肿瘤细胞的细胞质中，是肺癌组织中含量最丰富的肿瘤标记物，是非小细胞性肺癌较敏感的肿瘤标志物，尤其对肺鳞癌特异度高^[11]^。CA199多见于胰腺、肝胆和结直肠癌等恶性肿瘤的诊断中，但有研究^[7]^中出现CA199在肺癌组要明显高于良性疾病组及正常组（*P* < 0.05），这表明CA199在肺癌诊断中还是有一定价值。近来还有研究发现肺腺癌细胞能直接产生CEA、CA125、CA199等。

作为区域麻醉方法之一的椎旁神经阻滞已被广泛应用于肺癌手术患者。因此本研究选择椎旁神经阻滞用于胸腔镜肺癌根治术。

有研究^[12]^表明，手术应激使患者的交感神经兴奋，释放大量儿茶酚胺，儿茶酚胺通过β受体直接促进肿瘤的生长和转移，同时还通过对细胞免疫的抑制作用而间接促进肿瘤的生长与转移。应激反应对免疫功能的影响来自儿茶酚胺对淋巴细胞的直接作用，围手术期紧张焦虑、手术刺激、低体温、疼痛是应激反应的主要来源，控制这些因素可以有效降低应激反应。另外，阿片类镇痛药具有免疫抑制效应。阿片类药物可以通过多种渠道加速恶性肿瘤细胞的播散。它们可以刺激血管再生，后者是恶性肿瘤生长和播散的关键因素。此外，阿片类药物还可以部分激活环氧化酶-2，使得前列腺素E2分泌增加，由此刺激血管生成和肿瘤进展^[13]^。本研究术后静脉泵镇痛药物是地佐辛，地佐辛是一种对阿片受体兼有激动和拮抗作用的药物，主要是激动κ受体，对μ受体有拮抗作用，有研究^[14]^显示地佐辛用于结直肠癌患者术后镇痛对患者免疫功能影响较小。

本研究结果显示，GP组术后2 h和术后24 h的VAS值均明显低于G组，GP组患者各时点的VAS值非常低，镇痛完善; GP组术后24 h、48 h内启动自控按钮次数均明显少于G组; GP组24 h、48 h PCIA泵的输注总量均低于G组，这表明椎旁阻滞可有效减轻胸腔镜肺癌根治术患者术后疼痛并减少阿片类药物用量。另外，椎旁阻滞对术中镇痛药物瑞芬太尼的用量没有影响，两组比较差异无统计学意义。原因可能是用于椎旁神经阻滞的罗哌卡因达最大阻滞平面时间较慢25 min左右，在手术刺激强度较大时尚未达最大阻滞平面，术中主要依靠瑞芬太尼镇痛; 罗哌卡因的感觉阻滞作用时间10 h甚至更长，所以GP组术后镇痛药物用量少于G组。鉴于上述地佐辛对患者免疫功能影响较小，GP组和G组对患者影响的主要区别是GP组椎旁阻滞的镇痛效果。

最近研究^[15]^结果表明，局部麻醉可以减少应激反应，保护围手术期免疫功能，减少阿片类药物依赖，从而发挥有益作用，其机理可能是麻醉方法影响原发性癌症手术患者血浆中与血管生成相关因子的浓度，进而影响癌症的复发和转移。本研究结果与上述研究结果一致，但完善的围术期镇痛、阿片类药物和局部麻醉是否可以减少癌症患者应激反应、保护机体免疫功能并可能减少肿瘤的复发和转移有待进一步研究。

本研究表明，单纯全麻或胸椎旁神经阻滞复合全麻对胸腔镜肺癌根治术患者肿瘤标志物水平无明显影响。其可能的原因是：入选患者均为早期肺癌，有关早期肺癌肿瘤标志物是否升高及其意义尚有争议; 本研究的手术方式均为全腔镜肺癌根治术，与开胸手术相比，具有手术时间短，术中出血少，麻醉用药少，术后留置管道时间短等特点，可能并未达到影响肿瘤标志物的创伤程度。但两种麻醉方法是否影响到患者的免疫功能，乃至长期预后需要进一步大样本的回顾性分析甚至前瞻性研究。

本研究的缺陷在于，样本数较少，观察时间较短，所得出的结论是否能应用到创伤较大的胸科手术如食管癌手术需要进一步研究。

综上所述，胸椎旁神经阻滞可明显减轻胸腔镜肺癌根治术患者术后疼痛并减少静脉镇痛泵内阿片类药物用量; 胸椎旁神经阻滞对胸腔镜肺癌根治术患者肿瘤标志物水平无明显影响。
